# Histone deacetylase inhibitors modulate hormesis in leukemic cells with mutant FMS-like tyrosine kinase-3

**DOI:** 10.1038/s41375-023-02036-2

**Published:** 2023-09-21

**Authors:** Yanira Zeyn, Kristin Hausmann, Melisa Halilovic, Mandy Beyer, Hany S. Ibrahim, Walburgis Brenner, Siavosh Mahboobi, Matthias Bros, Wolfgang Sippl, Oliver H. Krämer

**Affiliations:** 1 Department of Toxicology, University Medical Center, 55131 Mainz, Germany; 2 Department of Dermatology, University Medical Center Mainz, Mainz, Germany; 3 Department of Medicinal Chemistry, Institute of Pharmacy, Martin-Luther-University of Halle-, Wittenberg, Halle (Saale) Germany; 4 Department of Pharmaceutical Chemistry, Faculty of Pharmacy, Egyptian Russian University, Badr City, Cairo Egypt; 5 Department of Obstetrics and Gynecology, University Medical Center Mainz, Mainz, Germany; 6 Institute of Pharmacy, Faculty of Chemistry and Pharmacy, University of Regensburg, 93040 Regensburg, Germany

**Keywords:** Cell signalling, Biochemistry

## To the Editor:

The FMS-like tyrosine kinase-3 (FLT3) is mutated in ~30% of acute myeloid leukemia (AML) patients. Common FLT3 mutations are internal tandem duplications (FLT3-ITD) and point mutations in its c-terminal tyrosine kinase domain (FLT3-TKD). The resulting active FLT3 receptors promote cell proliferation and resistance to programmed cell death (apoptosis) [1]. The poor prognosis of patients with FLT3-ITD has spurred an intensive search for FLT3 inhibitors (FLT3i) [2]. These include the nanomolar FLT3i quizartinib, which has produced promising benefits in AML patients with FLT3-ITD [3]. TKD mutations in FLT3-ITD arise during the therapy of patients with quizartinib and confer drug resistance. The broad-range tyrosine kinase inhibitors midostaurin and gilteritinib are used with standard chemotherapy to treat AML. Their pleiotropic actions frequently evoke hematological toxicity [1, 2]. Marbotinib is a hybrid FLT3i containing elements of quizartinib and bis(1H-indol-2-yl)methanone. Marbotinib specifically inhibits FLT3-ITD and FLT3-TKD [4, 5].

Epigenetic modifiers of the histone deacetylase (HDAC) family control the development and survival of blood cells. Compared to normal cells, certain leukemic cell types frequently have aberrant expression levels and activities of epigenetic modifiers that belong to the histone deacetylase (HDAC) family. Accordingly, inhibitors of zinc-dependent HDACs (HDACi) are approved drugs for the treatment of subtypes of leukemia and lymphoma. These agents are vorinostat, romidepsin, belinostat, and chidamide. Further HDACi are tested clinically (www.clinicaltrials.org), such as the benzamide entinostat (MS-275) [6, 7]. HDACi decrease FLT3-ITD through ubiquitin-dependent proteasomal degradation and apoptosis-associated caspase activation. Combinations of HDACi and FLT3i synergistically kill FLT3-ITD-positive cells through accelerated elimination of FLT3-ITD and DNA replication stress/DNA damage induction. HDAC1, HDAC2, and particularly HDAC3 maintain the stability of FLT3-ITD [8]. A cellular self-digestion process termed autophagy also eliminates FLT3-ITD [9]. Nonetheless, autophagy can promote FLT3i resistance [10, 11]. HDACs modulate autophagy in leukemic cells [6, 12], but it is unknown if HDACi modulate FLT3-ITD through autophagy and if this has biological relevance.

Despite initial hope in HDACi, their limited clinical efficacy as monotherapies and toxicological issues make additional research necessary. This includes the development of nanomolar HDACi with a low impact on normal cells and the identification of effective drug combinations [7, 13]. KH16 and KH29 are recently developed HDACi, which we have named yanostat-1/yanostat-2 [12, 14]. We reveal that their inhibitory profiles are superior to those of SAHA and MS-275 in AML cells carrying FLT3-ITD. Low doses of HDACi cause hormesis effects through FLT3-ITD. Specific inhibition of FLT3-ITD with the nanomolar FLT3i marbotinib and quizartinib abrogates undesired hormesis and is synergistically lethal in combination with nanomolar doses of KH16.

**Fig. 1:**
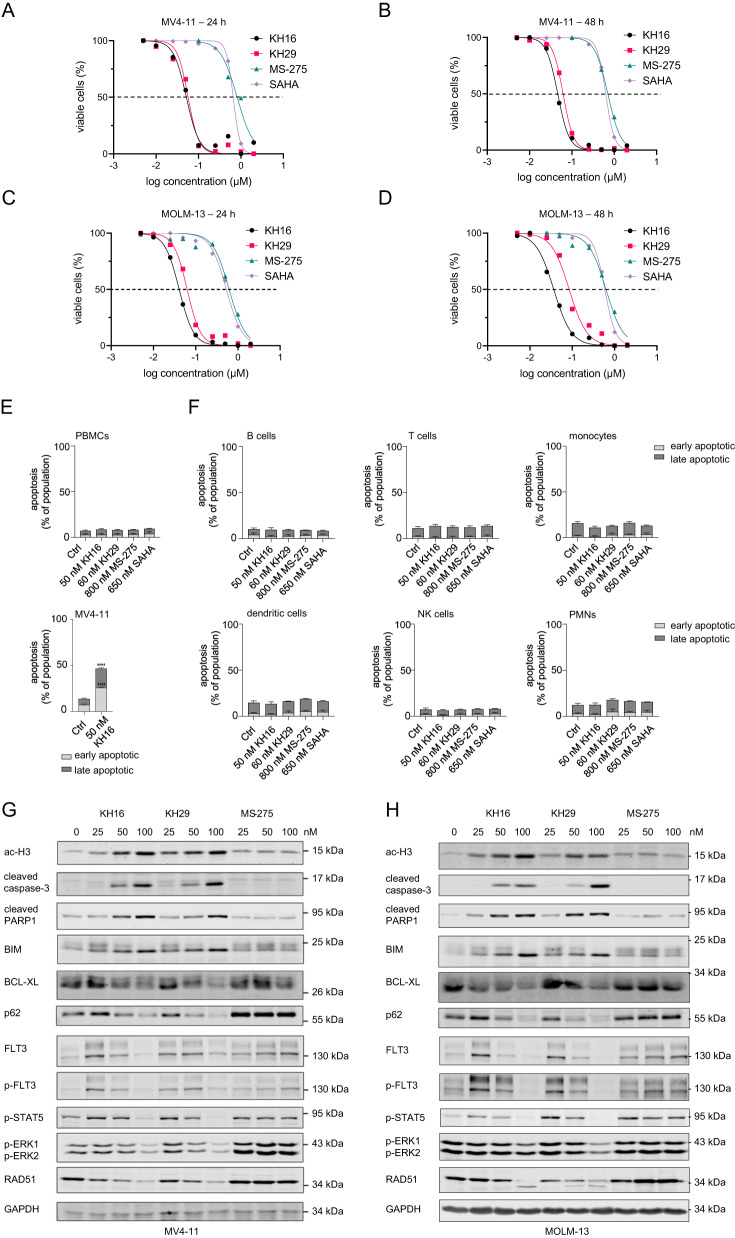
Cellular and biochemical effects of KH16 and KH29. MV4-11 and MOLM-13 cells were treated with different concentrations of KH16, KH29, MS-275, or SAHA for 24-48 h and subjected to flow cytometry analyses for annexin-V (indicator of early apoptosis; #130-093-060 from Miltenyi Biotec) and propidium iodide (PI; from Sigma-Aldrich; late apoptosis indicator). **A** MV4-11 cells were treated with increasing concentrations of KH16, KH29, MS-275, or SAHA from 5 nM to 2 µM for 24 h (*n* = 3). The x-axis was logarithmically transformed to yield a dose-response curve and to calculate the IC_50_ using GraphPad Prism 8.4.3. **B** Same experiments as in **A**, but for 48 h. Decreased cell viability equals to annexin-V/PI-positivity (*n* = 3). **C** MOLM-13 cells were treated with increasing concentrations of KH16, KH29, MS-275, or SAHA (5 nM to 2 µM) and incubated for 24 h (*n* = 3) or **D** Same experiments as in **C**, but for 48 h (*n* = 3). **E** Upper panel: PBMCs were isolated from buffy coats of four healthy donors (tested negative for common infections) using Biocoll (Bio&Sell). These were treated with HDACi as indicated for 24 h (Ctrl, untreated) and subjected to flow cytometry assessing apoptosis. To receive and delineate the PBMC populations, debris and doublets were excluded. Lower panel: MV4-11 cells that were treated in parallel to the PBMCs with 50 nM KH16 are a positive control for apoptosis induction (*n* = 4; mean+SD; two-way ANOVA; ****p ≥ 0.0001). **F** Untreated and HDACi-treated PBMCs were stained for lineage markers and subjected to flow cytometry (Ctrl, untreated). The cells were defined as: CD3^-^CD19^+^ as B cells; CD3^+^ as T cells, CD3^-^CD19^-^CD14^+^ as monocytes; CD3^-^CD19^-^CD1c^+^ as dendritic cells; CD3^-^CD19^-^CD56^+^ as NK cells; and CD3^-^CD14^-^CD19^-^CD56^-^CD11b^+^ as PMNs. The cell populations were analyzed for viability using annexin-V AF647 (#A23204; early apoptosis marker) and FVD eFl780 (#65-0865-18; late apoptosis marker; both from ThermoFisher). The following antibodies were used: CD11b BV510 (#101263), CD1c BV605 (#331538), CD3 BV711 (#344838) from BioLegend; CD14 PE-eFl610 (#61-0149-42), CD56 Pe-Cy7 (#25-0567-42), CD19 AF488 (#53-0199-42) from ThermoFisher. **G** MV4-11 cells were treated with 25, 50, and 100 nM KH16, KH29, or MS-275, respectively, for 24 h. Immunoblot was carried out for the indicated proteins, ac-H3, acetylated histone H3, GAPDH as representative loading control; *n* = 2. **H** MOLM-13 cells were treated with 25, 50, and 100 nM KH16, KH29, or MS-275, respectively for 24 h; *n* = 2. The *n*-numbers indicate biological replicates of which some were additionally carried out as technical replicates. Immunoblot was carried out for the indicated proteins, GAPDH as representative loading control; *n* = 2. Flow cytometry and immunoblot were done as mentioned [4, 12], with the following antibodies: BCL-XL (#ab32370), BIM (#ab32158), GAPDH (#ab128915), RAD51 (#ab6380) from Abcam; cleaved PARP (#552596) from BD Biosciences; FLT3 (#sc-480), SQSTM1/p62 (#sc-25575) from Santa Cruz Biotechnology; cleaved caspase-3 (#cs9661), p-Tyr591-FLT3 (#3461), p-Tyr202/Tyr204-ERK1/ERK2 (#cs9101) from Cell Signaling; p-Tyr694-STAT5 (#MA5-14973) from Thermo Fisher; ac-H3 (#06-599) from Millipore. The protein ladder is the prestained Scientific^TM^ PageRuler^TM^ (#26617) from Thermo Fisher.

KH16 and KH29 carry the hydroxamic acid as zinc binding moiety being present in SAHA and have nanomolar activity against HDACs [12]. We applied KH16 and KH29 to human FLT3-ITD-positive MV4-11 and MOLM-13 AML cells for 24-48 h. Flow cytometry demonstrated that nanomolar concentrations of KH16 and KH29 induced apoptosis (37 nM < IC_50_ < 85 nM). KH16 and KH29 were at least 6.3-fold more effective apoptosis inducers than the established HDACi MS-275 and SAHA (533 nM < IC_50_ < 814 nM) (Fig. 1A–D; Table 1; Fig. S1A, B). Thus, KH16 and KH29 are significantly more potent inducers of apoptosis in FLT3-ITD-positive leukemic cells than the clinical grade HDACi vorinostat and entinostat.

**Table 1:** KH16 and KH29 are more potent inducers of apoptosis than MS-275 and SAHA in FLT3-ITD-positive leukemic cells.

	KH16		KH29		MS-275		SAHA	
hours	24	48	24	48	24	48	24	48
MV4-11 (IC_50_ nM)	52	46	57	62	814	706	658	629
MOLM-13 (IC_50_ nM)	41	37	63	85	621	628	533	586

Shown are the IC_50_ values for apoptosis induction in MV4-11 and MOLM-13 cells that were treated with KH16, KH29, MS-275, or SAHA for 24-48 h. The values were calculated from the flow cytometry experiments (Fig. 1A–D) using GraphPad Prism 8.4.3; *n* = 3.

Human embryonic kidney and retinal pigment epithelial (RPE1) cells do not undergo apoptosis when they are exposed to KH16 and KH29 [12, 14]. We extended these analyses. We incubated MV4-11 cells, RPE1 cells, and human keratinocytes (HaCaT cells) with rounded IC_50_ concentrations of KH16, KH29, and SAHA for 24-72 h. Flow cytometry showed that the HDACi induced apoptosis significantly in MV4-11 cells but not in RPE1 and HaCaT cells (Fig. S2). To additionally consider the responses of normal human blood cells to KH16 and KH29, we isolated peripheral blood mononuclear cells (PBMCs) from healthy donors and treated the cells with rounded IC_50_ concentrations of KH16, KH29, MS-275, and SAHA for 24 h. None of these HDACi compromised the viability of PBMCs (Fig. 1E). We further distinguished the main leukocyte populations by employing antibodies for lineage-specific surface markers. We found that HDACi did not induce apoptosis in B-cells, T-cells, monocytes, dendritic cells, natural killer (NK) cells, and polymorphonuclear neutrophils (PMNs) (Fig. 1F). Thus, despite being potent inducers of apoptosis in leukemic cells with FLT3-ITD, KH16 and KH29 do not kill normal cells.

Immunoblot analyses with lysates from MV4-11 and MOLM-13 cells that were incubated with equimolar concentrations of KH16, KH29, and MS-275 verified that 25 nM of KH16 and KH29 induced an accumulation of hyperacetylated histone H3 (Fig. 1G, H). This effect increased dose-dependently. Up to 100 nM MS-275 did not induce an accumulation of acetylated histone H3.

We confirmed that KH16 and KH29 induced apoptosis by immunoblots assessing activation of caspase-3 and cleavage of its target PARP1. Moreover, we detected a dose-dependent induction of pro-apoptotic BIM and a reduction of anti-apoptotic BCL-XL. KH16 and KH29 caused these molecular alterations more effectively than MS-275. Whereas 25 nM KH16 and KH29 induced the autophagy protein p62, 100 nM KH16 and KH29 decreased p62. 25–100 nM MS-275 stabilized p62 (Fig. 1G, H).

Unexpectedly, 25–50 nM KH16 and KH29, and 25–100 nM MS-275, evoked an accumulation of total and phosphorylated FLT3-ITD (Fig. 1G, H). At 100 nM concentrations of KH16 and KH29, this effect was lost. This held for the phosphorylation of the kinases ERK1/ERK2, the transcription factor STAT5, and the levels of the DNA repair protein RAD51. Doses of 100 nM KH16 and KH29 attenuated these tumor-promoting targets of FLT3-ITD (Fig. 1G, H).

**Fig. 2:**
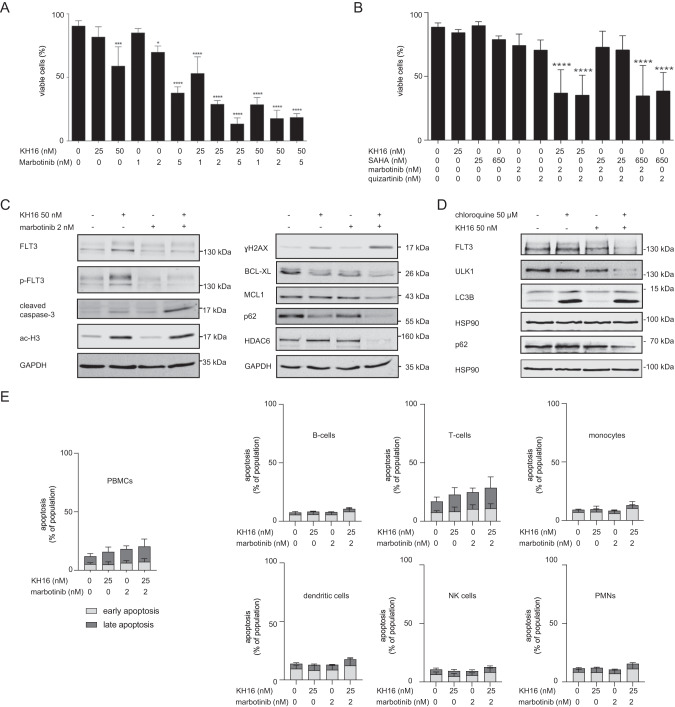
Combinations of KH16 and FLT3i efficiently kill FLT3-ITD-positive leukemic cells. **A** MV4-11 cells were treated with 25-50 nM KH16 ± 1-5 nM marbotinib for 24 h and subjected to flow cytometry for apoptosis measurement (*n* = 3; mean+SD; one-way ANOVA; *p ≥ 0.05; ***p ≥ 0.001; ****p ≥ 0.0001). **B** MV4-11 cells were treated with different combinations of HDACi and FLT3i as indicated for 24 h and subjected to flow cytometry for apoptosis measurement (*n* = 4; mean+SD; one-way ANOVA; ****p ≥ 0.0001) **C** Immunoblot shows the levels of the indicated proteins in MV4-11 cells that were treated as indicated for 24 h; GAPDH serves as loading control (*n* = 2). **D** MV4-11 cells were treated with 50 nM KH16 ± 50 µM chloroquine for 24 h. Immunoblot was carried out for the indicated proteins; HSP90, loading control (*n* = 2). The *n*-numbers indicate biological replicates of which some were additionally carried out as technical replicates. Flow cytometry and immunoblot were done as mentioned [4, 12], with the following antibodies: BCL-XL (#ab32370), GAPDH (#ab128915) from Abcam; FLT3 (#sc-480), HSP90 (#sc-13119), MCL-1 (#sc-12756), SQSTM1/p62 (#sc-25575), ɣH2AX (#sc-101696) from Santa Cruz Biotechnology; cleaved caspase-3 (#cs9661), HDAC6 (#7558), LC3B (#3868), p-Tyr591-FLT3 (#3461), ULK1 (#8054) from Cell Signaling; ac-H3 (#06-599) from Millipore. The protein ladder is the prestained Scientific^TM^ PageRuler^TM^ (#26617) from Thermo Fisher. **E** PBMCs from four healthy donors were isolated, treated as indicated, separated in cell fractions, and analyzed with flow cytometry (*n* = 4; mean+SD; see Fig. 1E, F for details).

We aimed to define the biological importance of the accumulation of p-FLT3-ITD and FLT3-ITD in response to low doses of KH16 (Fig. 1G, H). We hypothesized that this increase in FLT3-ITD attenuated pro-apoptotic effects of HDACi. We experimentally addressed this with a nanomolar dose of marbotinib. We found that 25 nM KH16 and 2 nM marbotinib combined favorably against MV4-11 cells (Fig. 2A). We also noted this effect using 25 nM KH16 and 2 nM quizartinib. Compared to KH16 plus FLT3i, combinations of 25 nM SAHA and the FLT3i were significantly less effective apoptosis inducers. In such combinations, increasing the dose of SAHA to 650 nM gave similar results as 25 nM KH16 (Fig. 2B). According to these results, combinations of KH16 plus marbotinib or quizartinib are low nanomolar inducers of apoptosis in FLT3-ITD-positive leukemic cell populations.

Immunoblot analyses verified that 50 nM KH16 plus 2 nM marbotinib activated caspase-3, induced the DNA damage marker ɣH2AX, and decreased the anti-apoptotic proteins BCL-XL and MCL1. Histone hyperacetylation was equally evoked by KH16 ± marbotinib (Fig. 2C). These findings suggest that low doses of HDACi cause hormesis through kinase signaling. KH16 plus marbotinib decreased the autophagy regulators p62 and HDAC6 (Fig. 2C), indicating a breakdown of autophagy. Inhibition of autophagy with chloroquine potentiated the ability of 50-100 nM KH16 to attenuate p62 and FLT3-ITD in MV4-11 and MOLM-13 cells. Moreover, this combination treatment caused a reduction and cleavage of the UNC-51-like autophagy-activating kinase-1 (ULK1) (Figs. 2D; S3). Caspase-dependent mechanisms likely conferred this loss and processing of proteins that belong to the autophagy system [12].

We analyzed how the combination of 25 nM KH16 and 2 nM marbotinib affected PBMCs and subpopulations therein. We noted that these drugs alone and in combination did not significantly induce apoptosis of B-cells, T-cells, monocytes, dendritic cells, NK cells, and PMNs (Fig. 2E). These data suggest that these inhibitors selectively kill leukemic cells carrying mutant FLT3 and spare normal blood cells.

In conclusion, the epigenetic drugs KH16 and KH29 are innovative lead compounds that deserve further consideration as treatment options for leukemia. One needs to consider the threshold concentrations at which HDACi evoke caspase-dependent apoptosis and abrogate cytoprotective autophagy. This finding stresses the need for HDACi that are effective at low nanomolar concentrations. Specific FLT3 kinase inhibitors disable undesired hormesis effects that HDACi cause. This allows such drug combinations to favorably combine against leukemic cells that carry the clinically unfavorable marker FLT3-ITD.

### Supplementary information

Supplemental figures

Zeyn_original blots_Submission

Authorship Change Approval

## Data Availability

The datasets that we generated during and analyzed during this study are available from the corresponding authors on reasonable request.
