# Supplementary material for: Histone deacetylase inhibitors modulate hormesis in leukemic cells with mutant FMS-like tyrosine kinase-3
Source: Leukemia. 2023 Sep 21;37(11):2319–23. doi: 10.1038/s41375-023-02036-2 (PMC10624624; doi:10.1038/s41375-023-02036-2)
Supplement: Supplementary file 1 — Supplemental figures [file 41375_2023_2036_MOESM1_ESM.pdf]

Supplemental Figure S1: individual data for Figure 1A-1D

A

MV4-11

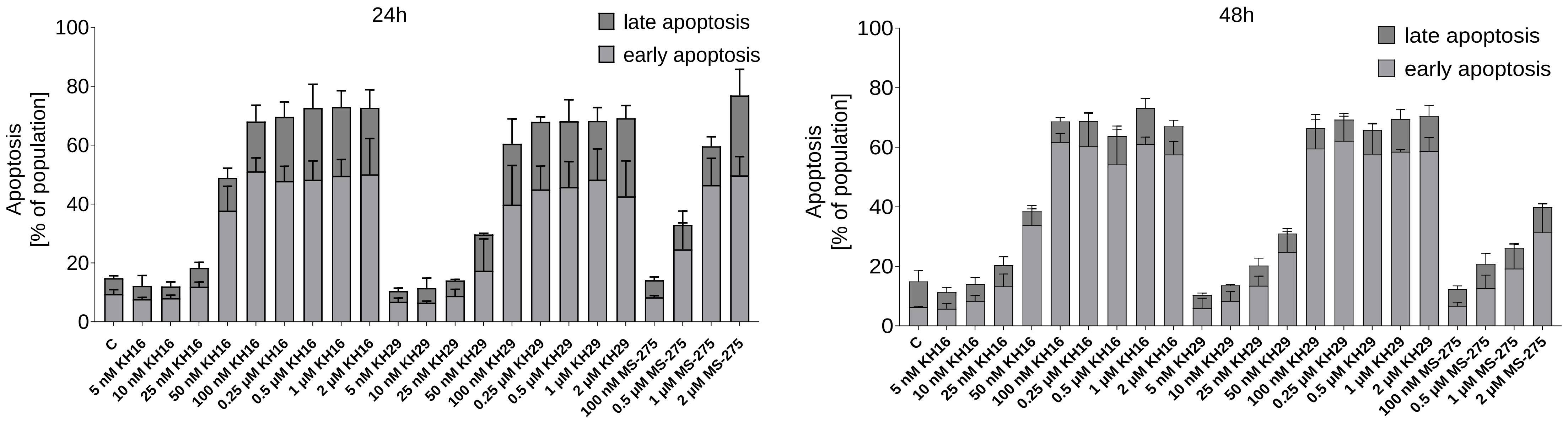

B

MOLM-13

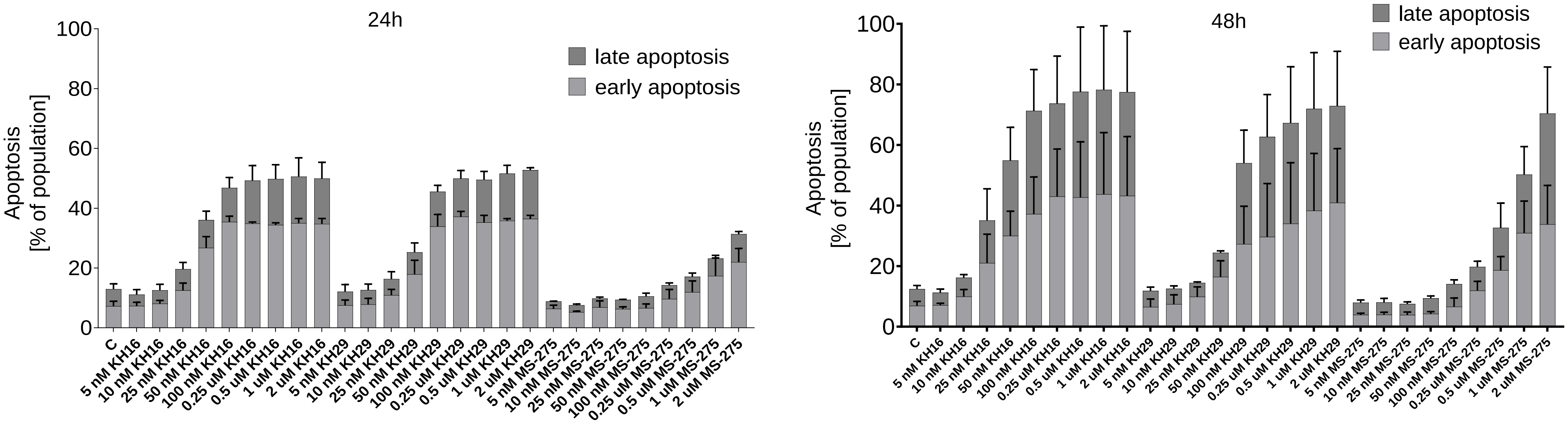

Supplemental Figure S2: pertinent to Figure 1A-F

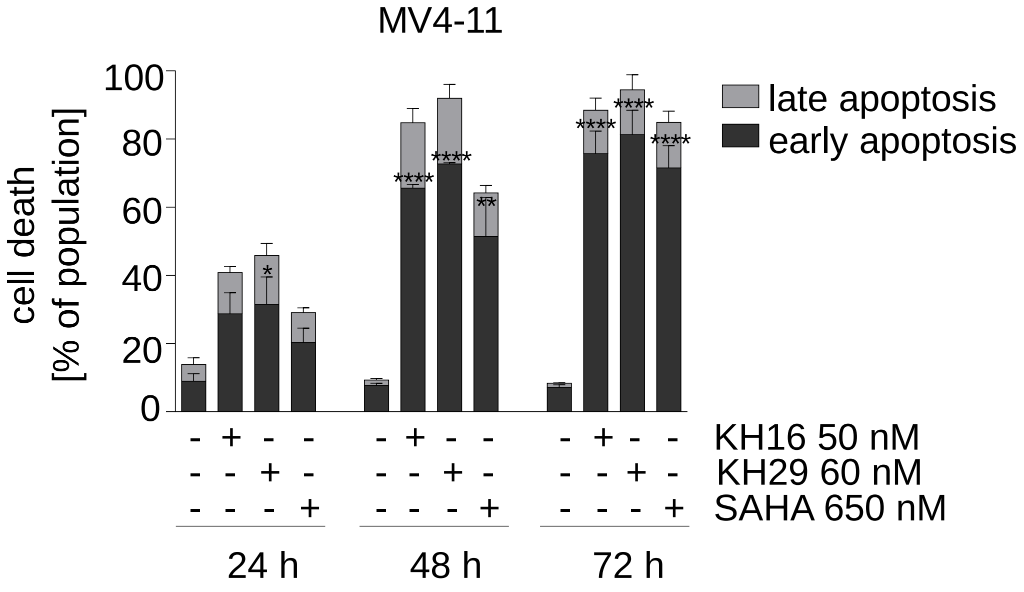

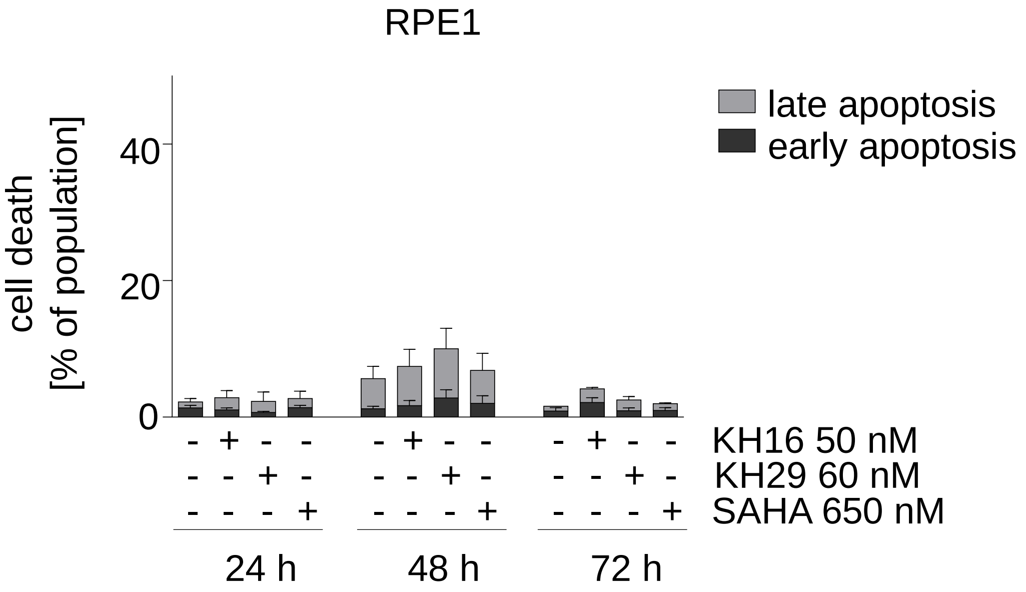

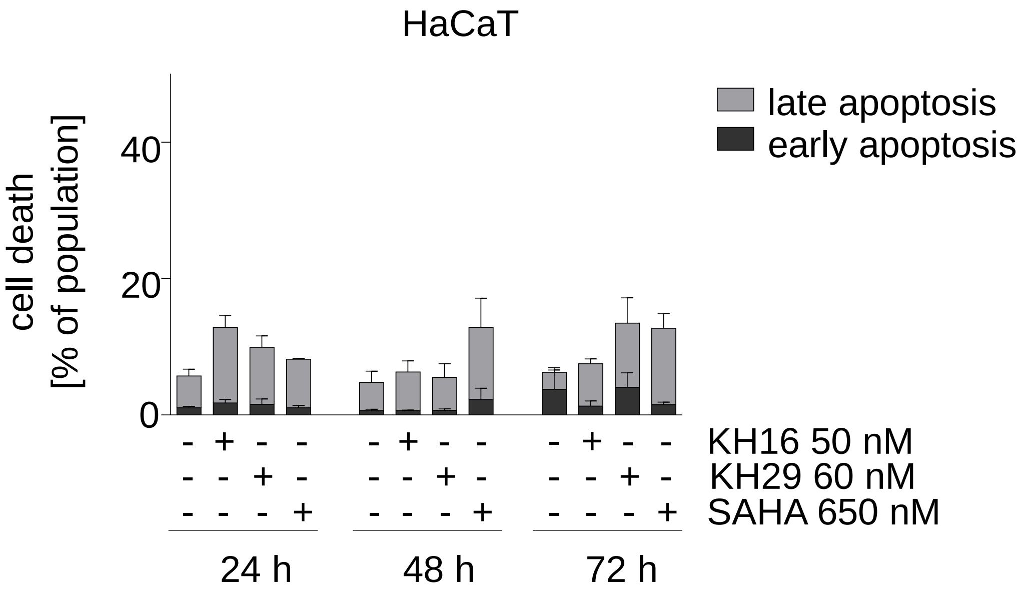

MV4-11, RPE1, and HaCaT cells were treated as indicated for 24-72 h. Cells were harvested and subjected to annexin-V (#130-093-060 from Miltenyi Biotec) and propidium iodide (PI) (Sigma-Aldrich) staining for apoptosis measurement by flow cytometry (n=3; mean+SD; two-way ANOVA; * p≥.0.05; *** p≥.0.001; **** p≥.0.0001). Cells were maintained at 37°C and 5% CO2 in a humidified atmosphere. The growth medium for MV4-11 and MOLM13 cell lines was RPMI-1640 medium and RPE1 and HaCaT cells were cultured in Dulbecco's Modified Eagle Medium (DMEM). Media were supplemented with 5% fetal calf serum (FCS, Sigma-Aldrich) and 1% penicillin/streptomycin (Sigma-Aldrich).

Supplemental Figure S3: pertinent to Figure 2D

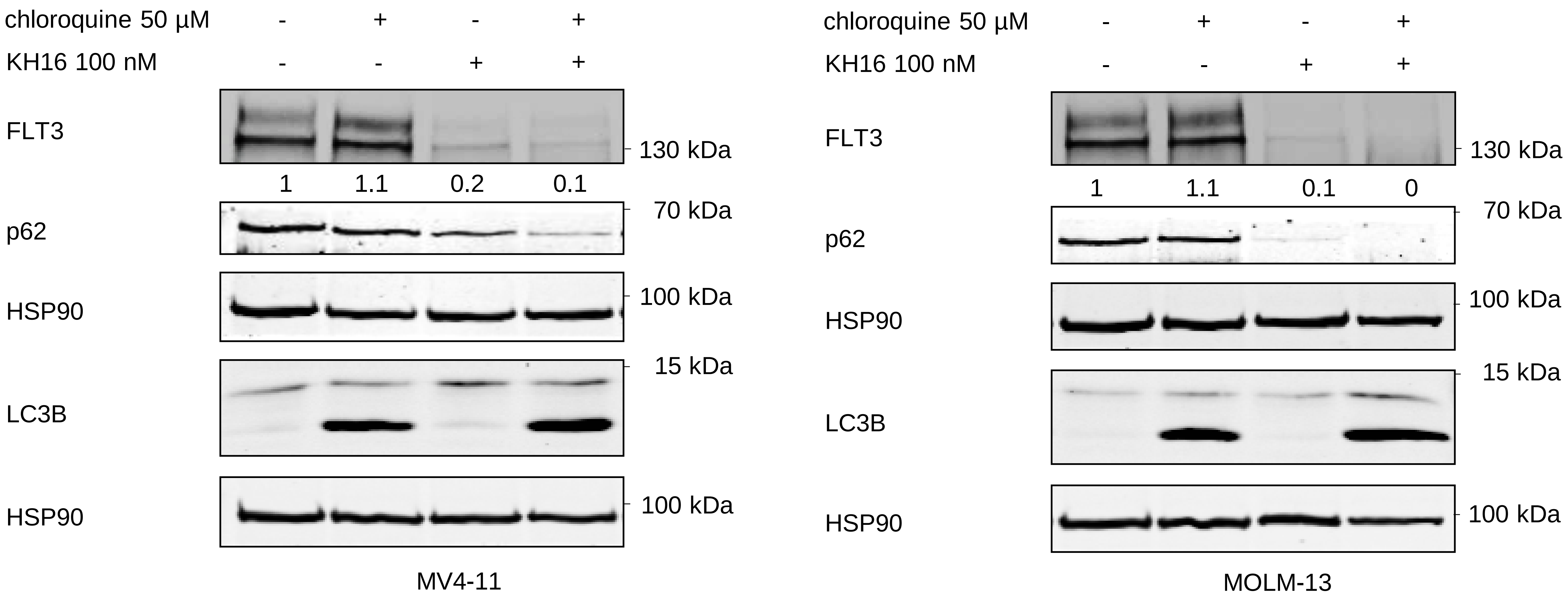

MV4-11 and MOLM-13 cells were treated with KH16 ± chloroquine for 24 h. Immunoblot was carried as indicated proteins; HSP90, loading control (n=2). Flow cytometry and immunoblot were done as mentioned (Beyer et al. 2022; Sellmer et al. 2020), with the following antibodies: BCL-XL (#ab32370), GAPDH (#ab128915), from Abcam; FLT3 (#sc-480), HSP90 (#sc-13119), MCL-1 (#sc-12756), SQSTM1/p62 (#sc-25575), ɣH2AX (#sc-101696) from Santa Cruz Biotechnology; cleaved caspase-3 (#cs9661), HDAC6 (#7558), LC3B (#3868), p-Tyr591-FLT3 (#3461) from Cell Signaling; ac-H3 (#06-599) from Millipore. The used protein ladder was the prestained Scientific™ PageRuler™ (#26617) from Thermo Fisher.
