# Supplementary material for: Histone deacetylase inhibitors modulate hormesis in leukemic cells with mutant FMS-like tyrosine kinase-3
Source: Leukemia. 2023 Sep 21;37(11):2319–23. doi: 10.1038/s41375-023-02036-2 (PMC10624624; doi:10.1038/s41375-023-02036-2)
Supplement: Supplementary file 2 — Zeyn_original blots_Submission [file 41375_2023_2036_MOESM2_ESM.pptx]

## Slide 1
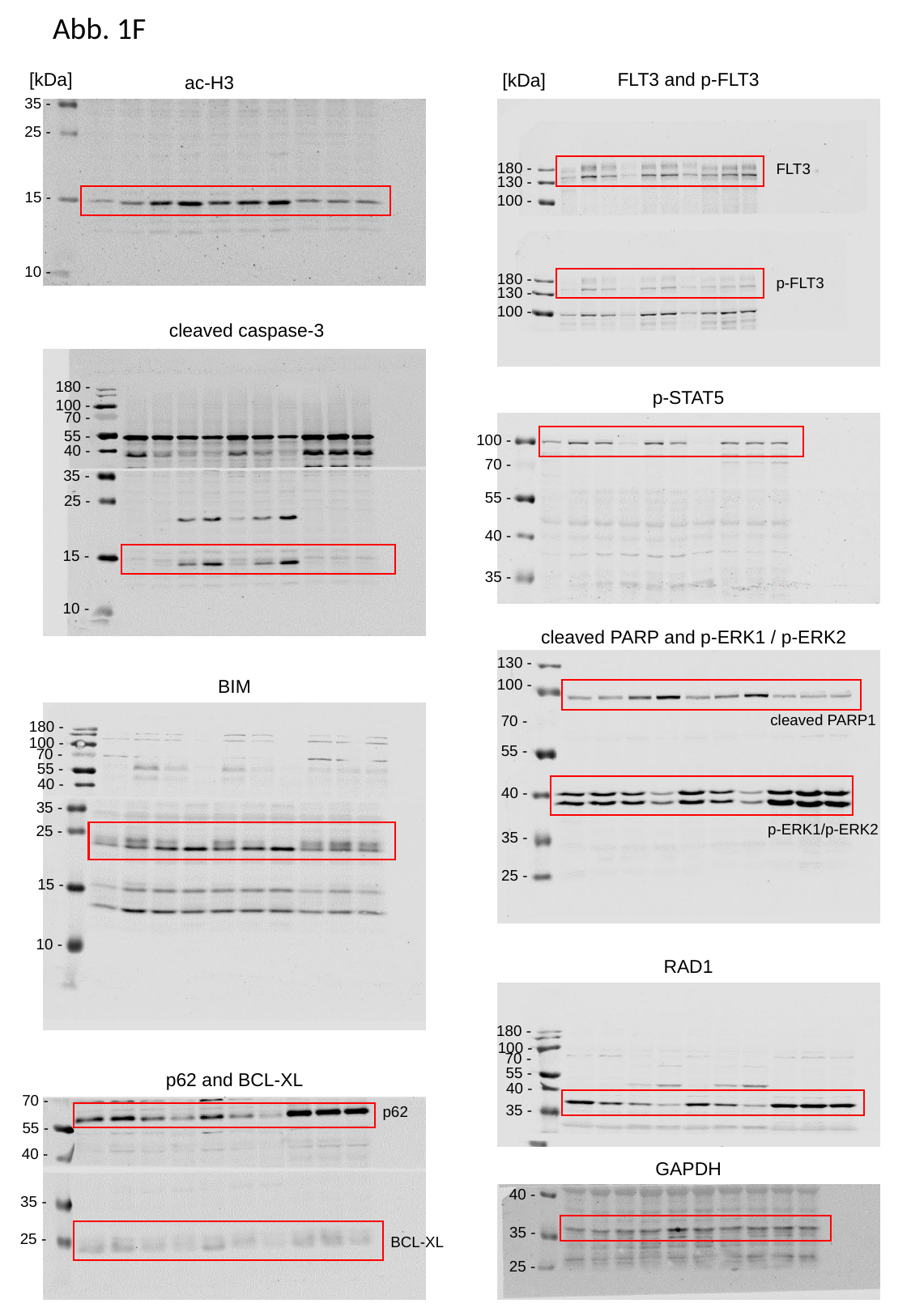

Abb. 1F
[kDa]
FLT3 and p-FLT3
[kDa]
ac-H3
35 -
25 -
180 -
FLT3
130 -
15 -
100 -
10 -
180 -
p-FLT3
130 -
100 -
cleaved caspase-3
180 -
p-STAT5
100 -
70 -
55 -
100 -
40 -
70 -
35 -
55 -
25 -
40 -
15 -
35 -
10 -
cleaved PARP and p-ERK1 / p-ERK2
130 -
BIM
100 -
cleaved PARP1
70 -
180 -
100 -
55 -
70 -
55 -
40 -
40 -
35 -
p-ERK1/p-ERK2
25 -
35 -
25 -
15 -
10 -
RAD1
180 -
100 -
70 -
55 -
p62 and BCL-XL
40 -
70 -
35 -
p62
55 -
40 -
GAPDH
40 -
35 -
35 -
25 -
BCL-XL
25 -

## Slide 2
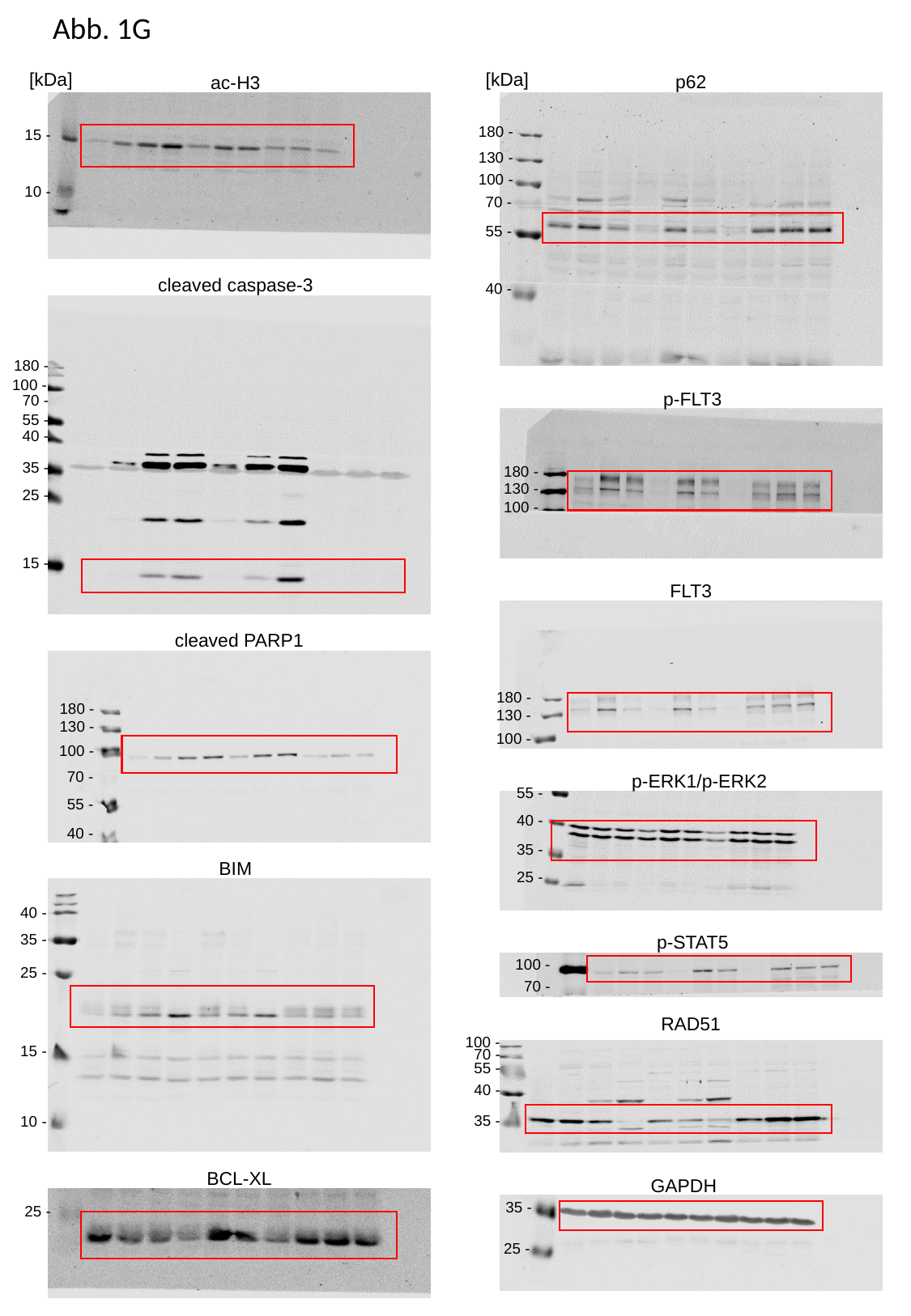

Abb. 1G
[kDa]
[kDa]
p62
ac-H3
180 -
15 -
130 -
100 -
10 -
70 -
55 -
cleaved caspase-3
40 -
180 -
100 -
p-FLT3
70 -
55 -
40 -
35 -
180 -
130 -
25 -
100 -
15 -
FLT3
cleaved PARP1
180 -
180 -
130 -
130 -
100 -
100 -
70 -
p-ERK1/p-ERK2
55 -
55 -
40 -
40 -
35 -
BIM
25 -
40 -
35 -
p-STAT5
100 -
25 -
70 -
RAD51
100 -
15 -
70 -
55 -
40 -
35 -
10 -
BCL-XL
GAPDH
35 -
25 -
25 -

## Slide 3
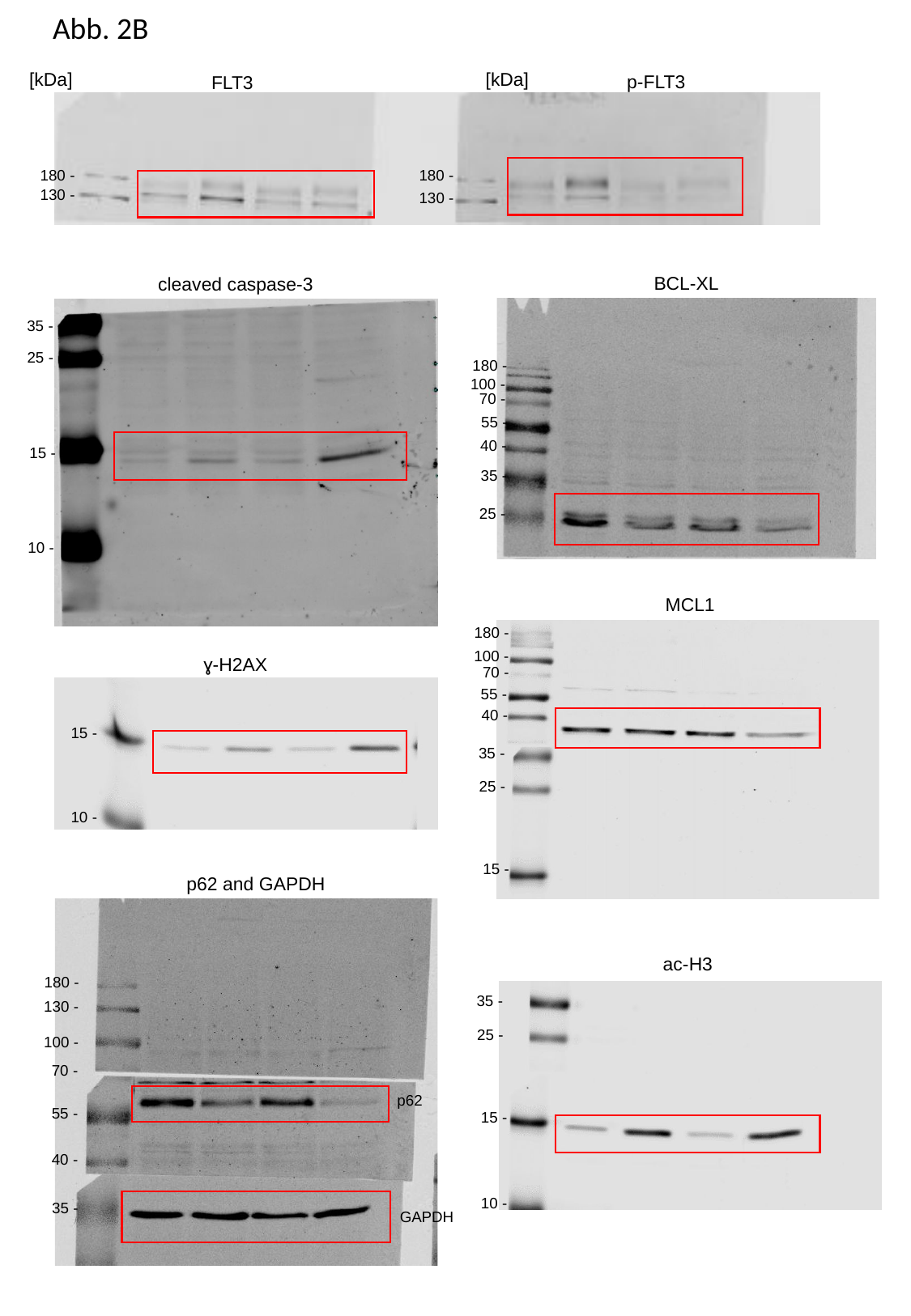

Abb. 2B
[kDa]
[kDa]
p-FLT3
FLT3
180 -
180 -
130 -
130 -
BCL-XL
cleaved caspase-3
35 -
25 -
180 -
100 -
70 -
55 -
40 -
15 -
35 -
25 -
10 -
MCL1
180 -
100 -
ɣ-H2AX
70 -
55 -
40 -
15 -
35 -
25 -
10 -
15 -
p62 and GAPDH
ac-H3
180 -
35 -
130 -
25 -
100 -
70 -
p62
55 -
15 -
40 -
10 -
35 -
GAPDH

## Slide 4
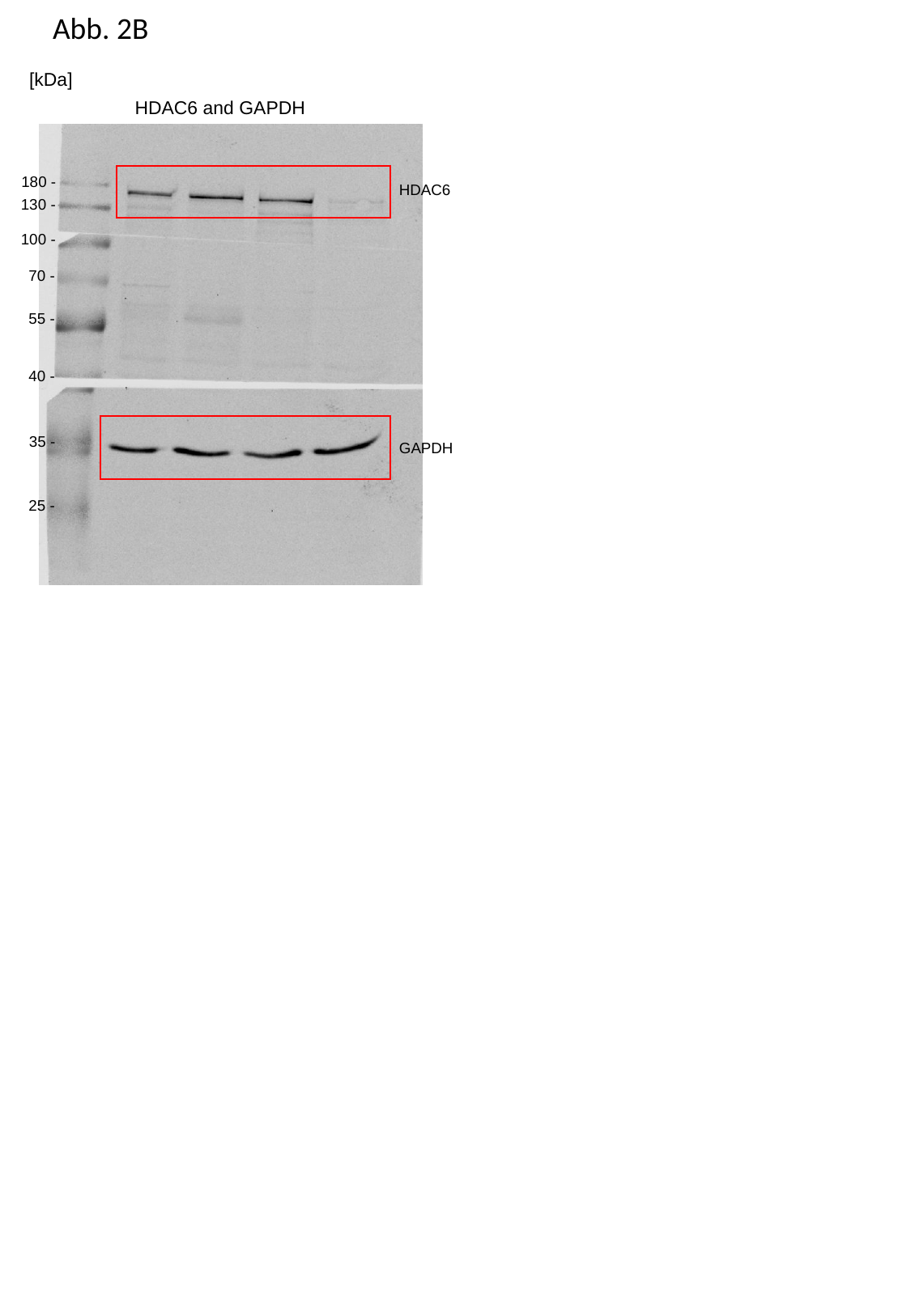

Abb. 2B
[kDa]
HDAC6 and GAPDH
180 -
HDAC6
130 -
100 -
70 -
55 -
40 -
35 -
GAPDH
25 -

## Slide 5
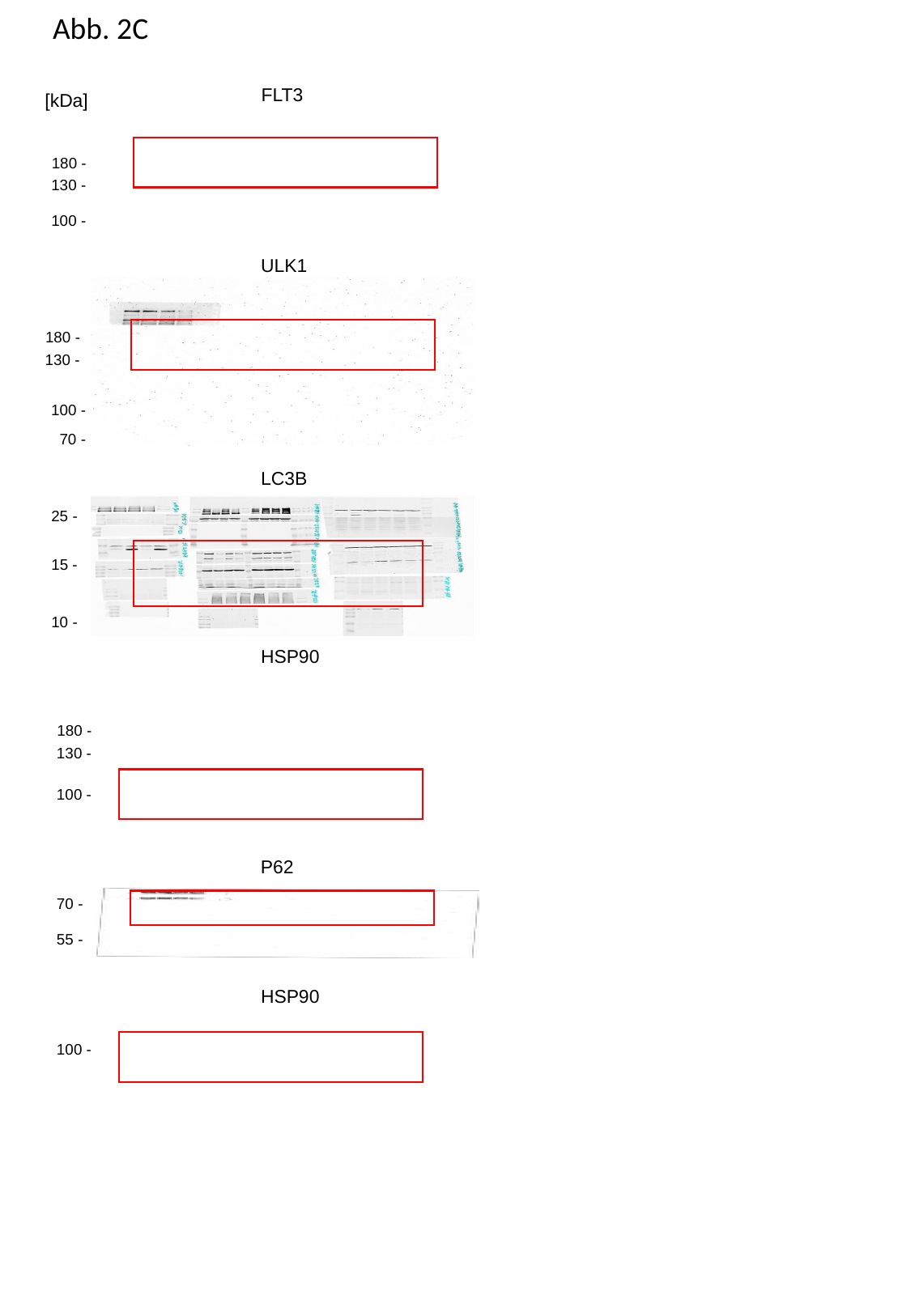

Abb. 2C
FLT3
[kDa]
180 -
130 -
100 -
ULK1
180 -
130 -
100 -
70 -
LC3B
25 -
15 -
10 -
HSP90
180 -
130 -
100 -
P62
70 -
55 -
HSP90
100 -
