# Supplementary material for: Histone deacetylase inhibitors modulate hormesis in leukemic cells with mutant FMS-like tyrosine kinase-3
Source: Leukemia. 2023 Sep 21;37(11):2319–23. doi: 10.1038/s41375-023-02036-2 (PMC10624624; doi:10.1038/s41375-023-02036-2)
Supplement: Supplementary file 3 — Authorship Change Approval [file 41375_2023_2036_MOESM3_ESM.pdf]

yanira.zeyn@uni-mainz.de

AW: Matthias Bros added in revision - Nachricht (HTML)

Datei Nachricht Hilfe

AW: Matthias Bros added in revision

Zeyn, Yanira
An Brenner, Prof. Dr. Walburgis; Beyer, Mandy
Cc Krämer, Oliver; Halilovic, Melisa; Bros, Dr. Matthias; Wolfgang Sippl; Siavosh Mahboobi; hany.ibrahim@pharmazie.uni-halle.de; kristin.hausmann@gmx.de

Do 07.09.2023 09:42

OK to me.

Yanira Zeyn
Doktorandin

UNIVERSITÄTSMEDIZIN
der Johannes Gutenberg-Universität Mainz
Hautklinik AG Grabbe/Bros PKZI 308A
Langenbeckstraße 1
D - 55131 Mainz

Telefon: +49 (0) 6131 17-9758
E-Mail: yanira.zeyn@uni-mainz.de

Von: Brenner, Prof. Dr. Walburgis
Gesendet: Mittwoch, 6. September 2023 23:11:25
An: Beyer, Mandy
Cc: Krämer, Oliver; Zeyn, Yanira; Halilovic, Melisa; Bros, Dr. Matthias; Wolfgang Sippl; Siavosh Mahboobi; hany.ibrahim@pharmazie.uni-halle.de; kristin.hausmann@gmx.de
Betreff: Re: Matthias Bros added in revision

I confirm my agreement.
Walburgis Brenner

Am 06.09.2023 um 21:04 schrieb Beyer, Mandy <manbeyer@uni-mainz.de>:

25°C Sonnig 11:27 07.09.2023

kristin.hausmann@gmx.de

Aw: Matthias Bros added in revision - Nachricht (HTML)

Datei Nachricht Hilfe

Aw: Matthias Bros added in revision

Kristin Hausmann <kristin.hausmann@gmx.de>
An Krämer, Oliver

Do 07.09.2023 10:42

I confirm

best regards
Kristin Hausmann

E-mail: kristin.hausmann@gmx.de

T +4916091770745

linkedin.com/in/kristin-hausmann-03a563262

Gesendet: Mittwoch, 06. September 2023 um 19:40 Uhr
Von: "Krämer, Oliver" <okraemer@uni-mainz.de>
An: "Zeyn, Yanira" <yanira.zeyn@uni-mainz.de>, "Halilovic, Melisa" <halilovm@uni-mainz.de>, "Bros, Dr. Matthias" <mbros@uni-mainz.de>, "Wolfgang Sippl" <wolfgang.sippl@pharmazie.uni-halle.de>, "Siavosh Mahboobi" <Siavosh.Mahboobi@chemie.uni-regensburg.de>, "Brenner, Prof. Dr. Walburgis" <brenner@uni-mainz.de>, "Beyer, Mandy" <manbeyer@uni-mainz.de>, "hany.ibrahim@pharmazie.uni-halle.de" <hany.ibrahim@pharmazie.uni-halle.de>, "kristin.hausmann@gmx.de" <kristin.hausmann@gmx.de>
Cc: "Krämer, Oliver" <okraemer@uni-mainz.de>
Betreff: Matthias Bros added in revision

Dear All,

Please respond to this email until tomorrow 12 noon. Needed is a reply confirming that you approve of the change: Matthias Bros has been added

Histone deacetylase inhibitors modulate hormesis in leukemic cells with mutant FMS-like tyrosine kinase-3

It has come to our attention that your current author list has changed since your initial submission: Matthias Bros has been added.
To confirm your revised author list, we require agreement from all co-authors. To provide this agreement, please email all authors and ask them to reply confirming that they approve of the change. Please combine screenshots of all responses into a single PDF file, and upload this with your submission. Please name this file 'Authorship Change Approval', and use the 'Related Manuscript File' file type.

Best wishes!

25°C Sonnig 11:26 07.09.2023

Halilovic, Melisa halilovm@uni-mainz.de

Re: Matthias Bros added in revision

Halilovic, Melisa
An Zeyn, Yanira; Brenner, Prof. Dr. Walburgis; Beyer, Mandy
Cc Krämer, Oliver; Bros, Dr. Matthias; Wolfgang Sippl; Siavosh Mahboobi; hany.ibrahim@pharmazie.uni-halle.de; kristin.hausmann@gmx.de

Do 07.09.2023 12:17

OK to me.

Melisa Halilovic

From: Zeyn, Yanira
Sent: Thursday, September 7, 2023 9:42:07 AM
To: Brenner, Prof. Dr. Walburgis; Beyer, Mandy
Cc: Krämer, Oliver; Halilovic, Melisa; Bros, Dr. Matthias; Wolfgang Sippl; Siavosh Mahboobi; hany.ibrahim@pharmazie.uni-halle.de; kristin.hausmann@gmx.de
Subject: AW: Matthias Bros added in revision

OK to me.

Yanira Zeyn
Doktorandin

UNIVERSITÄTSMEDIZIN
der Johannes Gutenberg-Universität Mainz
Hautklinik AG Grabbe/Bros PKZI 308A
Langenbeckstraße 1
D - 55131 Mainz

Telefon: +49 (0) 6131 17-9758
E-Mail: yanira.zeyn@uni-mainz.de

Von: Brenner, Prof. Dr. Walburgis
Gesendet: Mittwoch, 6. September 2023 23:11:25
An: Beyer, Mandy
Cc: Krämer, Oliver; Zeyn, Yanira; Halilovic, Melisa; Bros, Dr. Matthias; Wolfgang Sippl; Siavosh Mahboobi; hany.ibrahim@pharmazie.uni-halle.de; kristin.hausmann@gmx.de
[illegible]

Mandy Beyer manbeyer@uni-mainz.de

RE: Matthias Bros added in revision

Beyer, Mandy
An Krämer, Oliver; Zeyn, Yanira; Halilovic, Melisa; Bros, Dr. Matthias; Wolfgang Sippl; Siavosh Mahboobi; Brenner, Prof. Dr. Walburgis; hany.ibrahim@pharmazie.uni-halle.de; kristin.hausmann@gmx.de

Mi 06.09.2023 21:05

OK to me
Mandy Beyer

-------- Ursprüngliche Nachricht --------
Von: "Krämer, Oliver" <okraemer@uni-mainz.de>
Datum: 06.09.23 19:40 (GMT+01:00)
An: "Zeyn, Yanira" <yanira.zeyn@uni-mainz.de>, "Halilovic, Melisa" <halilovm@uni-mainz.de>, "Bros, Dr. Matthias" <mbros@uni-mainz.de>, Wolfgang Sippl <wolfgang.sippl@pharmazie.uni-halle.de>, Siavosh Mahboobi <Siavosh.Mahboobi@chemie.uni-regensburg.de>, "Brenner, Prof. Dr. Walburgis" <brenner@uni-mainz.de>, "Beyer, Mandy" <manbeyer@uni-mainz.de>, hany.ibrahim@pharmazie.uni-halle.de, kristin.hausmann@gmx.de
Cc: "Krämer, Oliver" <okraemer@uni-mainz.de>
Betreff: Matthias Bros added in revision

Dear All,

Please respond to this email until tomorrow 12 noon. Needed is a reply confirming that you approve of the change: Matthias Bros has been added

Histone deacetylase inhibitors modulate hormesis in leukemic cells with mutant FMS-like tyrosine kinase-3

It has come to our attention that your current author list has changed since your initial submission: Matthias Bros has been added.
To confirm your revised author list, we require agreement from all co-authors. To provide this agreement, please email all authors and ask them to reply confirming that they approve of the change. Please combine screenshots of all responses into a single PDF file, and upload this with your submission. Please name this file 'Authorship Change Approval', and use the 'Related Manuscript File' file type.

Best wishes!

hany.ibrahim@pharmazie.uni-halle.de

Re: Matthias Bros added in revision - Nachricht (HTML)

Datei Nachricht Hilfe

Löschen Archivieren Verschieben Antworten Allen antworten Weiterleiten Posteingang Als ungelesen markieren Suchen Zoom

Re: Matthias Bros added in revision

Hany Ibrahim <hany.ibrahim@pharmazie.uni-halle.de>
An Siavosh.Mahboobi@chemie.uni-regensburg.de; kristin.hausmann@gmx.de; Wolfgang Sippl; Brenner, Prof. Dr. Walburgis; Halilovic, Melisa; Beyer, Mandy; Bros, Dr. Matthias; Krämer, Oliver; Zeyn, Yanira

Mi 06.09.2023 19:51

Ok to me

Hany Ibrahim

>>> On 09/06/2023 at 07:40 PM, Krämer, Oliver <okraemer@uni-mainz.de> <okraemer@uni-mainz.de> wrote:
Dear All,

Please respond to this email until tomorrow 12 noon. Needed is a reply confirming that you approve of the change: Matthias Bros has been added

Histone deacetylase inhibitors modulate hormesis in leukemic cells with mutant FMS-like tyrosine kinase-3

It has come to our attention that your current author list has changed since your initial submission: Matthias Bros has been added.
To confirm your revised author list, we require agreement from all co-authors. To provide this agreement, please email all authors and ask them to reply confirming that they approve of the change. Please combine screenshots of all responses into a single PDF file, and upload this with your submission. Please name this file 'Authorship Change Approval', and use the 'Related Manuscript File' file type.

Best wishes!

22°C Stark bewölkt 21:28 06.09.2023

Brenner, Prof. Dr. Walburgis brenner@uni-mainz.de

Re: Matthias Bros added in revision - Nachricht (HTML)

Datei Nachricht Hilfe

Löschen Archivieren Verschieben Antworten Allen antworten Weiterleiten Posteingang Als ungelesen markieren Suchen Zoom

Re: Matthias Bros added in revision

Brenner, Prof. Dr. Walburgis
An Beyer, Mandy
Cc Krämer, Oliver; Zeyn, Yanira; Halilovic, Melisa; Bros, Dr. Matthias; Wolfgang Sippl; Siavosh Mahboobi; hany.ibrahim@pharmazie.uni-halle.de; kristin.hausmann@gmx.de

Mi 06.09.2023 23:11

I confirm my agreement.
Walburgis Brenner

Am 06.09.2023 um 21:04 schrieb Beyer, Mandy <manbeyer@uni-mainz.de>:

OK to me
Mandy Beyer

-------- Ursprüngliche Nachricht --------
Von: "Krämer, Oliver" <okraemer@uni-mainz.de>
Datum: 06.09.23 19:40 (GMT+01:00)
An: "Zeyn, Yanira" <yanira.zeyn@uni-mainz.de>, "Halilovic, Melisa" <halilovm@uni-mainz.de>, "Bros, Dr. Matthias" <mbros@uni-mainz.de>, Wolfgang Sippl <wolfgang.sippl@pharmazie.uni-halle.de>, Siavosh Mahboobi <Siavosh.Mahboobi@chemie.uni-regensburg.de>, "Brenner, Prof. Dr. Walburgis" <brenner@uni-mainz.de>, "Beyer, Mandy" <manbeyer@uni-mainz.de>, hany.ibrahim@pharmazie.uni-halle.de, kristin.hausmann@gmx.de
Cc: "Krämer, Oliver" <okraemer@uni-mainz.de>
Betreff: Matthias Bros added in revision

Dear All,

Please respond to this email until tomorrow 12 noon. Needed is a reply confirming that you approve of the change: Matthias Bros has been added

Histone deacetylase inhibitors modulate hormesis in leukemic cells with mutant FMS-like tyrosine kinase-3

It has come to our attention that your current author list has changed since your initial submission: Matthias Bros has been added.
To confirm your revised author list, we require agreement from all co-authors. To provide this agreement, please email all authors and ask them to reply confirming that they approve of the change. Please combine screenshots of all responses into a single PDF file, and upload this with your submission. Please name this file 'Authorship Change Approval', and use the 'Related Manuscript File' file type.

18°C Teilw. bewölkt 00:09 07.09.2023

Siavosh Mahboobi Siavosh.Mahboobi@chemie.uni-regensburg.de

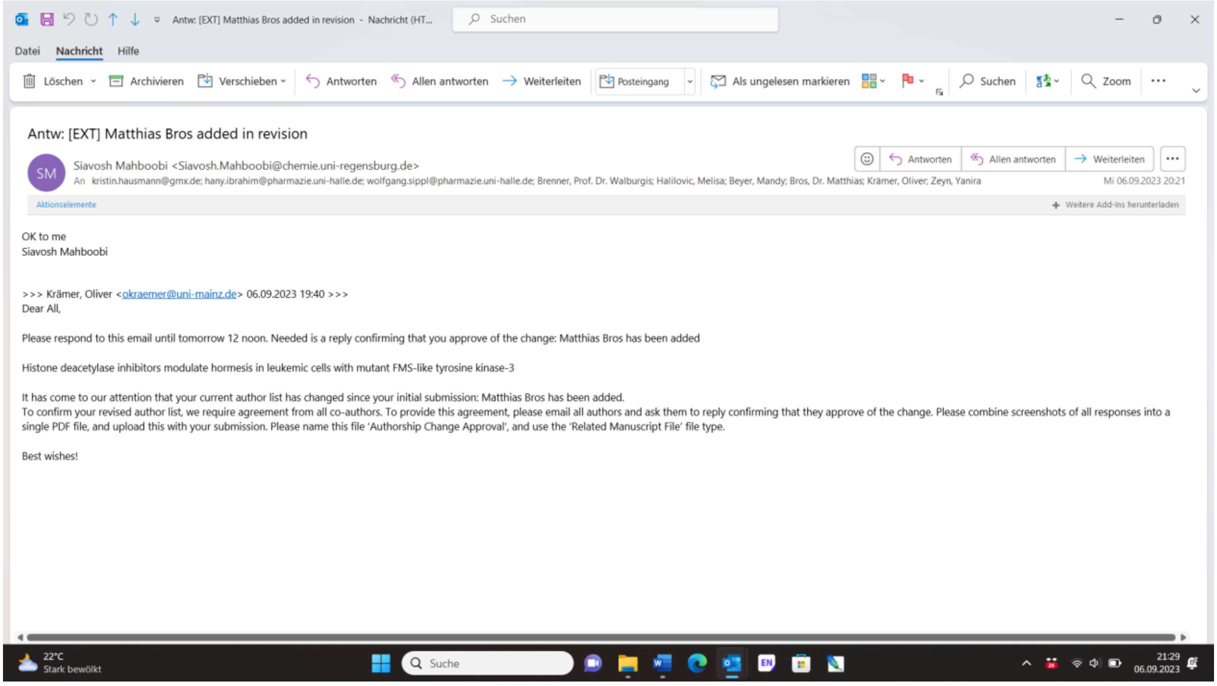

Antw: [EXT] Matthias Bros added in revision

Siavosh Mahboobi <Siavosh.Mahboobi@chemie.uni-regensburg.de>
An kristin.hausmann@gmx.de; hany.ibrahim@pharmazie.uni-halle.de; wolfgang.sippl@pharmazie.uni-halle.de; Brenner, Prof. Dr. Walburgis; Halilovic, Melisa; Beyer, Mandy; Bros, Dr. Matthias; Krämer, Oliver; Zeyn, Yanira
Mi 06.09.2023 20:21

OK to me
Siavosh Mahboobi

>>> Krämer, Oliver <okraemer@uni-mainz.de> 06.09.2023 19:40 >>>
Dear All,

Please respond to this email until tomorrow 12 noon. Needed is a reply confirming that you approve of the change: Matthias Bros has been added

Histone deacetylase inhibitors modulate hormesis in leukemic cells with mutant FMS-like tyrosine kinase-3

It has come to our attention that your current author list has changed since your initial submission: Matthias Bros has been added.
To confirm your revised author list, we require agreement from all co-authors. To provide this agreement, please email all authors and ask them to reply confirming that they approve of the change. Please combine screenshots of all responses into a single PDF file, and upload this with your submission. Please name this file 'Authorship Change Approval', and use the 'Related Manuscript File' file type.

Best wishes!

Bros, Dr. Matthias mbros@uni-mainz.de

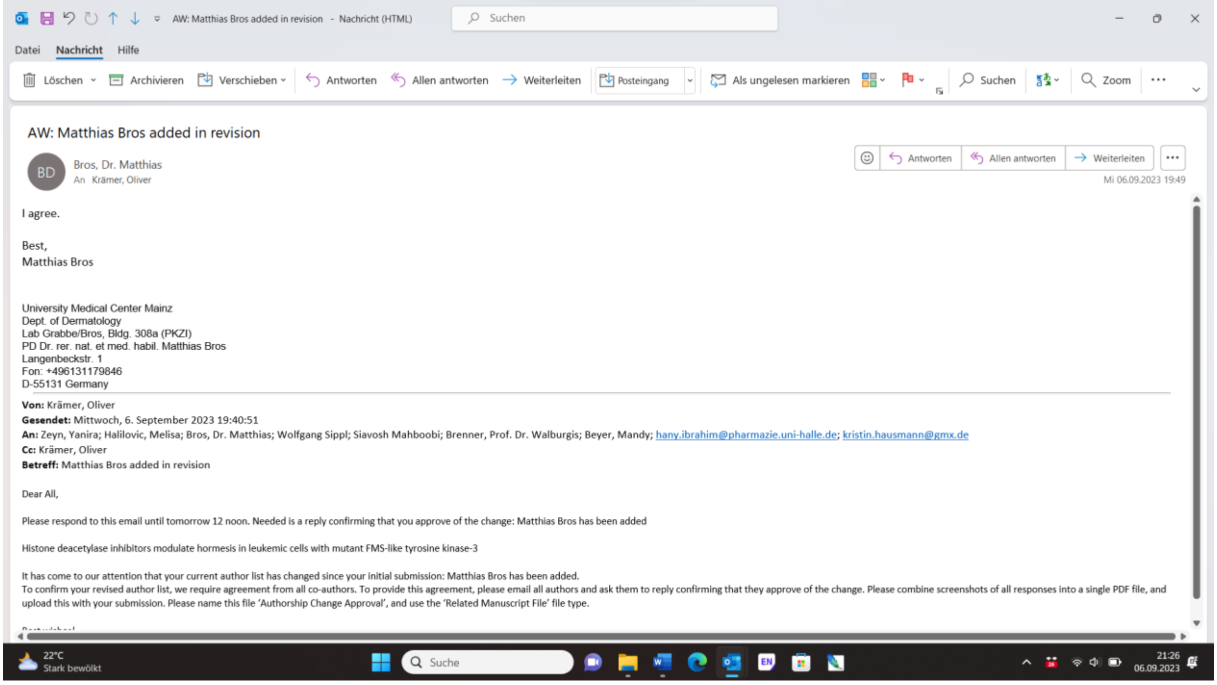

AW: Matthias Bros added in revision

Bros, Dr. Matthias
An Krämer, Oliver
Mi 06.09.2023 19:49

I agree.

Best,
Matthias Bros

University Medical Center Mainz
Dept. of Dermatology
Lab Grabbe/Bros, Bldg. 308a (PKZI)
PD Dr. rer. nat. et med. habil. Matthias Bros
Langenbeckstr. 1
Fon: +496131179846
D-55131 Germany

Von: Krämer, Oliver
Gesendet: Mittwoch, 6. September 2023 19:40:51
An: Zeyn, Yanira; Halilovic, Melisa; Bros, Dr. Matthias; Wolfgang Sippl; Siavosh Mahboobi; Brenner, Prof. Dr. Walburgis; Beyer, Mandy; hany.ibrahim@pharmazie.uni-halle.de; kristin.hausmann@gmx.de
Cc: Krämer, Oliver
Betreff: Matthias Bros added in revision

Dear All,

Please respond to this email until tomorrow 12 noon. Needed is a reply confirming that you approve of the change: Matthias Bros has been added

Histone deacetylase inhibitors modulate hormesis in leukemic cells with mutant FMS-like tyrosine kinase-3

It has come to our attention that your current author list has changed since your initial submission: Matthias Bros has been added.
To confirm your revised author list, we require agreement from all co-authors. To provide this agreement, please email all authors and ask them to reply confirming that they approve of the change. Please combine screenshots of all responses into a single PDF file, and upload this with your submission. Please name this file 'Authorship Change Approval', and use the 'Related Manuscript File' file type.

wolfgang.sippl@pharmazie.uni-halle.de

Re: Matthias Bros added in revision - Nachricht (HTML)

Re: Matthias Bros added in revision

Wolfgang Sippl <wolfgang.sippl@pharmazie.uni-halle.de>
An Krämer, Oliver
Cc Zeyn, Yanira; Halilovic, Melisa; Bros, Dr. Matthias; Siavosh Mahboobi; Brenner, Prof. Dr. Walburgis; Beyer, Mandy; Hany Ibrahim; Kristin Hausmann

Mi 06.09.2023 19:46

OK to me
Wolfgang Sippl

TypeApp for Android herunterladen
Am 6. Sept. 2023, um 19:40, "Krämer, Oliver" <okraemer@uni-mainz.de> schrieb:

> Dear All,
>
> Please respond to this email until tomorrow 12 noon. Needed is a reply confirming that you approve of the change: Matthias Bros has been added
>
> Histone deacetylase inhibitors modulate hormesis in leukemic cells with mutant FMS-like tyrosine kinase-3
>
> It has come to our attention that your current author list has changed since your initial submission: Matthias Bros has been added.
> To confirm your revised author list, we require agreement from all co-authors. To provide this agreement, please email all authors and ask them to reply confirming that they approve of the change. Please combine screenshots of all responses into a single PDF file, and upload this with your submission. Please name this file 'Authorship Change Approval', and use the 'Related Manuscript File' file type.
>
> Best wishes!

22°C Stark bewölkt — 21:25 06.09.2023

Oliver Krämer (last and corresponding author):

AW: Matthias Bros added in revision - Nachricht (Nur-Text)

AW: Matthias Bros added in revision

Krämer, Oliver
An Krämer, Oliver

Do 07.09.2023 17:16

I am more than happy that Matthias Bros has been included.

Best regards,

Oliver Krämer

-----Ursprüngliche Nachricht-----
Von: Krämer, Oliver <okraemer@uni-mainz.de>
Gesendet: Mittwoch, 6. September 2023 19:41
An: Zeyn, Yanira <yanira.zeyn@uni-mainz.de>; Halilovic, Melisa <halilovm@uni-mainz.de>; Bros, Dr. Matthias <mbros@uni-mainz.de>; Wolfgang Sippl <wolfgang.sippl@pharmazie.uni-halle.de>; Siavosh Mahboobi <Siavosh.Mahboobi@chemie.uni-regensburg.de>; Brenner, Prof. Dr. Walburgis <brenner@uni-mainz.de>; Beyer, Mandy <manbeyer@uni-mainz.de>; hany.ibrahim@pharmazie.uni-halle.de; kristin.hausmann@gmx.de
Cc: Krämer, Oliver <okraemer@uni-mainz.de>
Betreff: Matthias Bros added in revision

Dear All,

Please respond to this email until tomorrow 12 noon. Needed is a reply confirming that you approve of the change: Matthias Bros has been added

Histone deacetylase inhibitors modulate hormesis in leukemic cells with mutant FMS-like tyrosine kinase-3

It has come to our attention that your current author list has changed since your initial submission: Matthias Bros has been added.
To confirm your revised author list, we require agreement from all co-authors. To provide this agreement, please email all authors and ask them to reply confirming that they approve of the change. Please combine screenshots of all responses into a single PDF file, and upload this with your submission. Please name this file 'Authorship Change Approval', and use the 'Related Manuscript File' file type.

Best wishes!

29°C Sonnig — 17:16 07.09.2023
